# Exploring digital health care: eHealth, mHealth, and librarian opportunities

**DOI:** 10.5195/jmla.2021.1180

**Published:** 2021-07-01

**Authors:** Janet Chan

**Affiliations:** 1 jmz.reads@gmail.com, Research Assistant, University of South Florida, Tampa, FL

**Keywords:** eHealth, mHealth, librarian roles, digital health technology

## Abstract

Technology advances in eHealth and mHealth are changing the way that health care consumers and providers communicate, receive and deliver care, and access health information. As electronic health records and smartphones have become ubiquitous in the United States, opportunities and applications for the integration of eHealth and mHealth have increased. In addition to technology advances, the changing health care model is simultaneously adapting to and driving initiatives in digital health care. With these digital initiatives have come challenges, including data overload, security and privacy concerns, deficits in technological and health literacy skills, and sorting through the vast number of choices of digital applications. Navigating this changing landscape can be overwhelming and time consuming for both health care providers and consumers. Librarians are uniquely positioned to assist providers and consumers to break down barriers within the digital health care landscape through data management initiatives, technology and health literacy instruction, and finding and evaluating health information and digital health technologies.

Technology advances in eHealth and mHealth have had a marked effect on health care in the United States (US). These advances have given health care consumers and providers access to an array of new tools that impact the way in which health is monitored, information is exchanged, and records are maintained. However, these advances are not without their challenges. Digital tools produce an immense amount of data and require an aptitude in technology skills and digital literacy on the part of the user [1]. The ever-increasing number of digital health tools and applications have made sorting through the options time-consuming and at times overwhelming. Librarians are well positioned to help health care consumers and providers navigate this changing environment through data management support, teaching technology and health literacy skills, and locating and evaluating health information and digital health technologies. By applying their expertise in these areas, librarians play an important role in breaking down barriers to eHealth and mHealth initiatives within their communities.

## EHEALTH AND MHEALTH

Digital health care can be broadly separated into two categories: eHealth and mHealth. eHealth, “the use of information and communication technologies for health” [2], first arose in health care in the form of electronic health records (EHRs) [3]. EHRs consist of individual patient health information compiled during interactions within or across health organizations [1, 4]. The switch from paper to EHRs allowed clinicians new avenues of information access and clinical decision support [1]. Clinical records could now be accessed on computers from a variety of locations instead of being limited by a single paper chart kept in one location [5]. Clinical decision support tools were available at providers' fingertips and could be easily accessed while using the EHRs [1].

Although EHRs were identified as a future direction of health care as early as 1970, widespread adoption of EHRs did not increase significantly until the introduction of the Health Information Technology for Economic and Clinical Health (HITECH) Act in 2009 [1, 5]. After implementation of the HITECH Act, EHR adoption increases were seen across medical settings, with some of the greatest gains observed in clinical office settings [1]. Current adoption rates are at 96% in hospitals and 86% in office settings [6]. The most prevalent EHR systems in use in the United States are Epic, MediTech, and Cerner [7]. These electronic records are data-rich sources that go beyond clinical management of patients and can also be used to improve quality performance in health care organizations and contribute to medical research efforts [1, 7]. As these systems have become a ubiquitous part of health care, expanded use and interoperability with other digital health care tools such as personal health records (PHRs) have been undertaken. PHRs are health information records maintained by individuals. Utilization of a PHR can be an effective way for patients to organize their health information and share it with health care providers. PHRs are now available from a variety of sources, including insurance companies, health care organizations, and private companies and can be independent or tethered to EHRs [8, 9]. PHRs have become part of the eHealth spectrum as electronic versions have replaced print as consumers' preferred format [1]. Approximately 43% of PHRs are available on mobile platforms [8], and some have interoperability with mobile health apps [3]. This integration of technologies is just one example of the innovative tools available through the latest avenue of exploration in the digital health field of mHealth.

mHealth, the “medical and public health practice supported by mobile devices, such as mobile phones, patient monitoring devices, personal digital assistants (PDAs), and other wireless devices” [10] is becoming ubiquitous due to the popularity of mobile devices and technology advances in digital health care. The widespread availability and affordability of Internet and cellular service has expanded the customer base for smartphones, computers, and wearables [11]. A 2019 Pew research survey [12] indicates that smartphone ownership has risen to 81%, with an increasing number of people using their smartphones for online activities. The mobile app market in 2017 offered over 325,000 apps for health care [13], with apps focused on consumer health and fitness among the most popular [1]. Combining mobile health apps and wearables presents consumers with even more powerful tools to interact with their health information. Tech companies have combined miniature bodily sensors with gamification and rewards to create wearables that are comfortable and engaging for consumers [14]. With the miniaturization of sensors, expanded monitoring is attainable for a wide range of conditions. Mobile health apps and wearables are used for a variety of purposes, including monitoring blood pressure and diabetes [1, 3]. “Smart slippers” can monitor gait and assess fall risk, and electrocardiogram recordings can detect cardiac conditions [3]. The interoperability of these data collection devices with the EHR can provide valuable information to health care providers about patients' health. With over 400 devices on the market that are EHR compatible, digital consumer health monitoring is a growing trend [15].

## CHANGING HEALTH CARE LANDSCAPE

The ability to collect health data virtually combined with a shifting health care model contributes to the momentum advancing digital health care. The health care model is evolving from a paternalistic hospital/office-centered model to a patient participatory model where services are increasingly delivered through telemedicine and remote monitoring [1, 16, 17]. This changing health care model is driving digital health care advances as consumers seek information, access to their care records, and alternative methods of patient-provider communication. The Health Information National Trends Survey [18] reveals that 72% of US adults obtained health information from electronic sources in the last 12 months. Additionally, 52% of US adults want to access their health records and interact with health care providers through secure online sources [19]. The Meaningful Use criteria in the HITECH Act incentivizes hospitals and care providers who offer these resources to patients [1, 20]. One common way that health care providers offer these services is through EHR patient portals [20, 21]. Patient portals are designed to allow patients access to their health test results, messaging, and appointment scheduling with providers [1]. Librarians have also engaged with these systems to give consumers access to reliable health information [1]. Providing reputable health information resources to patients empowers them to make informed decisions in partnership with their health care providers. Additionally, services are increasingly being rendered at lower costs outside of traditional hospital/office settings [16]. Patients can receive personalized care in their home setting through remote monitoring of common conditions like hypertension, asthma, and diabetes [1]. For patients who are geographically distant or have difficulty traveling, utilizing remote monitoring and telemedicine can be an especially valuable option for connecting with their providers and being active participants in their care [1, 22]. While these forces are driving advances in digital health care, challenges to eHealth and mHealth initiatives, including data overload, low health literacy, lack of technology skills, and provider knowledge deficits present difficulties that need to be addressed for full potential of these services to be realized. Librarians possess proficiencies that make them ideal contributors to the growing field of digital health care. Their expertise in data management, information searching and evaluation, and literacy instruction are just a few of the skills that librarians can utilize to address barriers impeding eHealth and mHealth initiatives.

## CHALLENGES AND LIBRARIAN OPPORTUNITIES

### Data management

The data collected by eHealth and mHealth initiatives needs to be mined, analyzed, and used in meaningful ways to make positive contributions to the health care industry [1]. As wearable device popularity and mobile app development have soared, the amount of data collected has created a problem for providers and device function [23]. Research identifies data overload as a barrier to providers adopting mHealth technologies [12, 23, 24]. Dinh-Le et al. found that “providers experience alert fatigue in their daily clinical decision support systems” from integration of wearable data into the EHR [13]. Kauw et al. identified the need for a “triage process” to filter out irrelevant data, which could be carried out by support staff or computer algorithms [24]. Loncar-Turukalo et al. acknowledged the need for reducing the amount of data collected to “reduce power consumption and latency” in wearables [23]. Having too much information to filter through and potential lags in processing relevant data impeded its meaningful use by providers.

Current and emerging roles for librarians' data management skills have been cited throughout the literature [25]. Data collected through the integrations of EHRs, PHRs, wearables, and mobile apps can be mined and analyzed for use in direct clinical applications, research, or to inform internal health care organization practices [7]. EHRs can be assets to research efforts as patient registries and data sources [1, 25]. Librarians can support researchers in their efforts by teaching data management skills and best practices and assisting with the development of data management plans. One initiative at the University of Massachusetts Medical School included the health sciences librarian as a valued member of the research team who developed a data dictionary and data request form [25]. Gleason noted that librarians can also assist researchers in mHealth with the organization and management of large volumes of data generated by mobile devices [11].

National library organizations now provide professional development opportunities for librarians participating in data management. For librarians who need to update or advance their data management skills, continuing education opportunities are available through the Network of the National Library of Medicine (NNLM) in data visualization, bioinformatics, research data management, and many other topics [26]. The American Library Association features a guide entitled “Keeping up with research data management” that features training modules along with tools and real-life examples of data management by librarians [27]. The Research Data Management Librarian Academy is an online Canvas course developed by librarians to introduce fellow information professionals to data management skills and to guide them in introducing data management services in their own institutions [28].

### Information searching and evaluating

Librarians are expert searchers and evaluators of information assets. These skills sets are useful in the selection and delivery of health information. One of the most noted examples of librarians' involvement in eHealth is providing information resources through EHRs [20, 21, 29, 30]. The St. Louis Children's Hospital integrates library consults for patient education through its Epic EHR system [30]. Clinical staff can initiate a personalized consult for educational resources, and the librarian fulfills the request and delivers the resources through the EHR system. Another health care organization that utilizes Epic EHR is Michigan Medicine at the University of Michigan [31]. Michigan Medicine established a collaboration among a librarian, clinicians, and the information technology (IT) department to direct patients to reliable online health resources. Librarians developed a database of educational resources and now maintain and update it in addition to marketing the resources to patients [31]. Louisiana State University Health Shreveport uses the Epic systems PHR component, My Chart, to connect patients to online health information. Through a partnership with their IT department and the timely release of the NNLM initiative Medline Plus Connect, librarians educate patients in the use of My Chart and are able to showcase the health education resources of Medline Plus Connect [21]. Hospital librarians at the Shepherd Center's Noble Learning Resource center were involved with the creation of a patient portal for the center [20]. Their responsibilities first revolved around creating a user guide for the portal, but after realizing its educational potential, librarians were able to incorporate the Learning Resource Centers' assets into the online portal to enhance the availability of educational resources to patients [20]. These initiatives showcase different approaches to providing reliable educational resources to consumers using the EHR systems. Librarians' knowledge of both freely available (e.g., Medline Plus Connect) or institutional subscription-based health resources makes them valuable assets in facilitating patient access to health information.

Although these examples highlight the role of health science librarians in providing health information, public librarians may be more accessible to patients needing health information. In an effort to assist librarians practicing outside a health environment, health science librarians from the Tompkins-McCaw Library for the Health Sciences produced educational classes designed to promote consumer health information skills to librarians [32]. These classes included finding and evaluating mobile health apps and distinguishing between consumer health apps and medical apps for clinical use [32]. As over 58% of smartphone users have downloaded a health app, and there are over 325,000 health apps available to choose from, public librarians who can assist in sorting through this plethora of choices to provide reliable recommendations provide a valuable service to consumers [13, 32].

Consumers are not the only ones who need help sorting through the vast amount of health apps. Providers need knowledge of mobile apps not only to prescribe to consumers but also to access professional health information resources on their mobile devices [33, 34]. Providers have expressed reservations about recommending mobile heath apps to patients [33]. Two areas highlighted in the literature are the lack of research studies to support prescribing mobile health apps and a lack of provider knowledge about which apps are reliable and effective [33, 35]. Terry noted as of 2015, there were only 260 research studies on the clinical use of mobile apps in the literature [35]. More recently, however, researchers appear to be addressing this deficit. A January 2021 search of PubMed revealed that over 400 articles containing randomized controlled trials of clinical mobile apps were indexed between 2015 and 2021. Despite these advances, Byambasuren et al. found the most common reason given by general practitioners for not prescribing mobile health apps continues to be a lack of knowledge of effective apps [33]. Lake stated that providers wanted help in identifying apps for consumer use as researching and evaluating mobile apps can be a time-consuming task that may hamper their ability to implement mHealth initiatives [14]. Fortunately, librarians can assist in these endeavors.

Librarians are well positioned to support providers' information needs regarding mobile apps. Providers indicate that they prefer to receive information on mobile apps through asynchronous online training videos, reading materials, or webinars [33]. Librarians can accomplish this through the use of LibGuides or online videos embedded within their library's webpage. The University of Utah Health spearheaded an interdisciplinary U-Bar initiative in 2015 that provided face-to-face services for consumers who were interested in or had been prescribed mobile health apps [13]. Librarians had several roles in this initiative, including developing an evaluation protocol for mobile health apps, creating an order entry within the EHR so providers could prescribe apps to patients, and coordinating the app review process for the team [13]. This initiative addressed both the information needs of the providers and consumers in this organization.

### Health literacy and technology skills

Utilizing eHealth and mHealth tools requires adequate health literacy and technology skills on the part of the consumer [1, 36]. A lack of these skills can hamper efforts to engage with eHealth and mHealth tools, including using PHRs and choosing mobile health apps [1, 37]. Using a PHR requires patients to accurately document their health history and to understand the medical terminology used within the record system [38]. Hemsley et al. noted that PHR use was adversely affected by low health literacy and computer skills [36]. Researchers also found that mobile health app use positively correlates with eHealth literacy [39]. This is significant because, as of 2018, an estimated “65% of consumer health care transactions with health care organizations will be mobile and 70% of health care organizations worldwide will invest in consumer-facing apps, wearables, remote health monitoring, and virtual care” [13]. As the health care system continues to become increasingly digitized, consumers lacking adequate health literacy and technology skills may find navigating the health care system increasingly difficult.

As only 12% of US adults have proficient health literacy skills, library programming that provides consumers an opportunity to develop these skills will help facilitate the use of digital health care tools [40]. NNLM offers a health literacy guide and training sessions for librarians interested in offering health literacy programming to their consumers [41]. Educating consumers in health literacy can decrease the disparities in the health care system by enhancing consumer understanding of health care conditions and promoting active participation in their health care.

Even with appropriate health literacy, privacy and security concerns about using technology that transmits or stores sensitive data online can hamper digital health adoption. These concerns are not unfounded, as the US averages 40 health care data breeches per month with a median breech size of over 6,500 records [42]. These breeches affect millions of Americans annually [42]. IT specialists in health care organizations and their health information exchange partners work to ensure the highest levels of information security through a technique called “Defense in Depth” [1]. Access control, physical restriction, encryption, and firewalls are a few of the defenses used to combat cyber criminals and protect patient's health information [1].

Information from EHRs and PHRs is not the only source of health care data breeches. Mobile apps and wearables transit and store personal health information across wireless networks making them susceptible to cyber-attacks [1, 15, 43]. As devastating as an information attack can be to a patient's privacy, attacks on apps and wearables that provide biomedical monitoring of critical bodily functions can be disastrous [15]. Although there has not been a documented attack on a personal medical device in the US, the Food and Drug Administration [44] has issued cybersecurity alerts on various devices including insulin pumps, cardiac pacemakers, telemetry monitors, and infusion pumps. Cybersecurity guidelines for both device manufacturers and health care organizations have been developed to reduce the risks to patients [44]. Librarians can educate consumers in the areas of privacy and security, health literacy, and technology skills to help ensure that vulnerable populations are not left behind in digital health initiatives.

The 2017 New Media Consortium Horizons report [45] identified digital literacy as a solvable challenge and one that libraries can address by offering programming on current technologies and privacy and security considerations in the online environment. Educating consumers about privacy and security of PHRs and mobile health tools will address barriers to eHealth and mHealth usage and create a more informed digital technology user. Consumers who can gain experience in using current technology through library programming will be able to better participate in the increasingly digital health care landscape.

## CONCLUSION

The digital wave of health care technologies has impacted the practice and delivery of health care. Health care consumers and providers have experienced data overload and knowledge deficits related to these technologies. Librarians are already providing support and bridging knowledge gaps in data management, health literacy, and technology skills. As eHealth and mHealth tools continue to multiply and evolve, librarians must stay abreast of these technologies so that they may continue to assist providers and consumers in their evaluation and use. Librarians are well equipped and positioned within the community to identify needs and implement initiatives required to address the changing nature of health care technologies. By continuing to look forward, librarians will remain essential contributors in breaking down the barriers within eHealth and mHealth for consumers and providers.
